# An In-Depth Study on the Inhibition of Quorum Sensing by *Bacillus velezensis* D-18: Its Significant Impact on *Vibrio* Biofilm Formation in Aquaculture

**DOI:** 10.3390/microorganisms12050890

**Published:** 2024-04-29

**Authors:** Luis Monzón-Atienza, Jimena Bravo, Silvia Torrecillas, Antonio Gómez-Mercader, Daniel Montero, José Ramos-Vivas, Jorge Galindo-Villegas, Félix Acosta

**Affiliations:** 1 Grupo de Investigación en Acuicultura (GIA), Instituto Ecoaqua, Universidad de Las Palmas de Gran Canaria, 35001 Las Palmas de Gran Canaria, Spain; luis.monzon@ulpgc.es (L.M.-A.); silvia.torrecillas@irta.cat (S.T.); antonio.gomez@fpct.ulpgc.es (A.G.-M.); jose.ramos@uneatlantico.es (J.R.-V.); 2 Aquaculture Program, Institut de Recerca i Tecnologia Agroalimentáries (IRTA), Centre de Sant Carles de la Rápita (IRTA-SCR), 43540 Sant Carles de la Rápita, Spain; 3 Research Group on Foods, Nutritional Biochemistry and Health, Universidad Europea del Atlántico, 39010 Santander, Spain; 4 Deparment of Genomics, Faculty of Biosciences and Aquaculture, Nord University, 8026 Bodø, Norway; jorge.galindo-villegas@nord.no

**Keywords:** *Bacillus*, quorum sensing, quorum quenching, biofilm, *Vibrio*, aquaculture

## Abstract

Amid growing concerns about antibiotic resistance, innovative strategies are imperative in addressing bacterial infections in aquaculture. Quorum quenching (QQ), the enzymatic inhibition of quorum sensing (QS), has emerged as a promising solution. This study delves into the QQ capabilities of the probiotic strain *Bacillus velezensis* D-18 and its products, particularly in *Vibrio anguillarum* 507 communication and biofilm formation. *Chromobacterium violaceum* MK was used as a biomarker in this study, and the results confirmed that *B. velezensis* D-18 effectively inhibits QS. Further exploration into the QQ mechanism revealed the presence of lactonase activity by *B. velezensis* D-18 that degraded both long- and short-chain acyl homoserine lactones (AHLs). PCR analysis demonstrated the presence of a homologous lactonase-producing gene, ytnP, in the genome of *B. velezensis* D-18. The study evaluated the impact of *B. velezensis* D-18 on *V. anguillarum* 507 growth and biofilm formation. The probiotic not only controls the biofilm formation of *V. anguillarum* but also significantly restrains pathogen growth. Therefore, *B. velezensis* D-18 demonstrates substantial potential for preventing *V. anguillarum* diseases in aquaculture through its QQ capacity. The ability to disrupt bacterial communication and control biofilm formation positions *B. velezensis* D-18 as a promising eco-friendly alternative to conventional antibiotics in managing bacterial diseases in aquaculture.

## 1. Introduction

Aquaculture is a vital industry with global significance, providing a substantial source of protein worldwide. However, the imposition of forced practices on aquatic ecosystems can induce stress in fish, detrimentally impacting aquaculture production [1]. Due to their sensitivity to stress factors, fish are highly susceptible to bacterial diseases [2], notably *Vibrio* spp. This vulnerability is a significant concern, considering that *Vibrio* species are responsible for causing vibriosis, a major epizootic disease that affects a broad spectrum of both wild and cultured fish species on a global scale [3]. The clinical presentation of vibriosis in fish encompasses lethargy, anorexia, exophthalmia, hemorrhages, ulcerations, and congestion in internal organs [4].

*Vibrio* spp. have evolved to employ biofilm production as a survival strategy in response to the diverse challenges posed by the aquatic environment [5]. Biofilm is characterized by a self-produced matrix of extracellular polymeric substances and serves as a bacterial sessile-building mechanism, providing protection against environmental conditions and various agents [6,7]. The formation of biofilm is intricately linked to infection, virulence, and pathogenicity, emphasizing the role of biofilm in bacterial adaptation and resilience [8]. Furthermore, biofilm formation enhances bacterial resistance to different agents, including antibiotics, thereby complicating the effectiveness of conventional treatment methods [7]. Paradoxically, while antibiotics remain the primary treatment for bacterial infections in aquaculture, their indiscriminate use has led to an alarming increase in multidrug-resistant bacteria, necessitating a shift in research focus towards alternative control methods [9].

Bacteria, as remarkable communicators, produce, release, and sense extracellular signaling molecules, facilitating interaction with their environment [10,11]. When the concentration of those signaling molecules reaches a critical threshold, known as a “quorum”, bacteria adjust their gene expression to elicit a specific response [12]. This process of cell-to-cell communication, which can occur both within and between species, is referred to as “quorum sensing” (QS) [10]. QS plays a pivotal role in various bacterial activities, including adhesion, biofilm formation, stress adaptation, and the production of virulence factors [13]. The extracellular signaling molecules involved in QS are termed “autoinducers” (AIs) [14]. There are various types of AIs, with N-acyl homoserine lactones (AHLs) being the primary AI for Gram-negative bacteria [15,16].

Recently, the disruption or inhibition of QS has emerged as a viable strategy to counteract the challenges caused by bacteria [17]. This process, known as quorum quenching (QQ), involves the use of chemical or enzymatic means to inhibit QS [16]. The various forms of QQ include the degradation of AI molecules, the inhibition of AI synthesis, and the blocking/inhibition/competition of AI binding to receptors [11,18]. QQ offers a unique perspective compared to conventional treatments, which primarily focus on bacterial growth inhibition. Through QQ, pathogenic bacteria can be transformed into harmless microorganisms, effectively eliminating their pathogenicity [11,19]. As such, QQ serves as an eco-friendly and effective alternative to antibiotics and other chemical control agents for managing bacterial diseases [20] and offers a potential solution to multidrug-resistant pathogens [7].

Remarkably, the disruptive capacity of QQ extends to bacteria classified as probiotics [21]. Probiotics, defined as “live microorganisms that, when administered in adequate amounts, confer a health benefit on the host” [22], have demonstrated several benefits in aquaculture. These include immunomodulation, increased utilization of digestive enzymes, and improvements in both gut health and water quality [23]. *Bacillus* spp. are among the most widely used probiotic bacteria in aquaculture [24] and exhibit the capacity to degrade AHLs, underscoring their potential as effective agents in managing bacterial diseases [25,26]. As the scientific community strives to address the challenges in aquaculture sustainably, understanding the intricate interplay between forced practices, bacterial communication, and innovative control strategies becomes paramount in shaping the future of this vital industry.

We successfully isolated and characterized the *Bacillus velezensis* D-18 strain in our prior work [27]. We subsequently delved into its advantageous effects on the innate immune status of European seabass in our last study [28]. Despite these advancements, the intricate interaction dynamics between this probiotic bacterium and potential pathogens remain uncertain. Consequently, drawing from the existing literature, the primary objective of the present study was to assess the QQ potential of *B. velezensis* D-18. We also aimed to explore its practical application as a biofilm disruptor specifically targeting *Vibrio* spp. and further enhance our understanding of the multifaceted roles played by *B. velezensis* D-18, particularly in disrupting *Vibrio anguillarum* 507 biofilms, thereby contributing valuable insights to the broader field of probiotic research and aquaculture management.

## 2. Materials and Methods

### 2.1. Bacterial Strains

The probiotic strain *Bacillus velezensis* D-18, previously isolated and characterized in our laboratory in prior studies, underwent routine cultivation via two methods: culturing on Luria–Bertani (LB) broth at 26 °C overnight or on brain–heart infusion (BHI) broth with 1.5% NaCl supplementation, following a standardization procedure. Bacterial growth was meticulously monitored at 3, 6, 9, 12, and 24 h using optical density measurements (OD600) and serial dilutions at each time point. These standardization procedures were performed in triplicate to ensure the reliability and reproducibility of the observed growth patterns.

The fish pathogenic strain isolated within our laboratory (*Vibrio anguillarum* 507) was routinely cultured at 26 °C on BHI broth with 1.5% NaCl supplementation. This process adhered to the following standardization: Similar to the *Bacillus velezensis* study, the growth of *Vibrio anguillarum* 507 was monitored at key time points (3, 6, 9, 12, and 24 h) using optical density measurements (OD600) and serial dilutions. This iteration of the experiment was also conducted in triplicate to ensure robustness and consistency in the results.

*Chromobacterium violaceum* MK, a wild-type strain (CECT494, obtained from the Spanish Type Culture Collection—CECT) producing quorum sensing (QS)-dependent purple pigment violacein, served as a key component in bioassays. This strain was routinely cultured on LB broth at 26 °C overnight.

The biosensor strain *Chromobacterium violaceum* CV026, a mini-Tn5 mutant of the wild-type ATCC31532 deficient in QS-dependent violacein production (from our laboratory collection), was employed to detect exogenous AHLs. *C. violaceum* CV026 produces the purple pigment violacein in response to short-chain AHLs and was cultured on LB broth at 26 °C overnight.

*Chromobacterium violaceum* VIR24 was provided by Instituto de Investigación Marqués de Valdecilla (IDIVAL, Santander, Cantabria) and was used as a biosensor to detect exogenous long-chain AHLs due to its production of violacein. The strain was routinely cultured on LB broth at 26 °C overnight.

*Bacillus subtilis* subsp. *subtilis* CECT39 (ytnP—homolog lactonase) and *Bacillus cereus* CECT148 (aiiA—lactonase) were utilized as control strains for lactonase genes. These strains were sourced from the Spanish Type Culture Collection (CECT) (Paterna, Spain) and were routinely cultured on LB broth at 37 °C overnight.

### 2.2. Quorum Quenching Assay

A quorum quenching assay was carried out in accordance with the protocol outlined in [21], with specific adjustments. Initially, two overnight cultures were initiated: one featuring *B. velezensis* D-18 at 26 °C and 140 rpm in LB broth and the other with *C. violaceum* MK under identical conditions. Then, 1.5 mL of the *B. velezensis* culture was subjected to centrifugation (14,000 rpm, 10 min), and the resulting supernatant was filtered through a 0.22 mm membrane to isolate extracellular products (ECPs). Simultaneously, the *B. velezensis* pellet was resuspended in 1.5 mL of PBS. Following this, 1 mL of ECPs and 1 mL of the *B. velezensis* culture were subjected to heat inactivation (99 °C/15 min). A culture medium was prepared by combining 1 mL of *C. violaceum* MK broth with 49 mL (1:50) of LB soft agar (0.4%), which was thoroughly mixed, agitated, and poured into 6-well plates. Once solidified, 10 µL of the *B. velezensis* culture, the *B. velezensis* pellet, ECPs, heat-inactivated *B. velezensis*, heat-inactivated ECPs, and PBS were added to each respective plate. The 6-well plates were then cultured for 24 h at 26 °C, and the entire experiment was conducted in triplicate to ensure experimental repeatability.

### 2.3. AHL Degradation by Bacillus velezensis D-18

The degradation of short- and long-chain AHLs (C6 and C12 AHLs, respectively) by *B. velezensis* D-18 was assessed with a methodology inspired by Santos et al. [29], with certain refinements. A single colony from a freshly cultivated and uncontaminated *B. velezensis* was cultured overnight in 25 mL of LB at 26 °C with continuous agitation at 140 rpm. From this 25 mL culture, 10 mL was subjected to centrifugation (12,000 rpm, 15 min, 4 °C), and the supernatant was filtered through a 0.2 µm membrane to obtain ECPs, some of which were also separated and tested to prevent any interference with violacein production by the biomarkers. The resulting pellet was resuspended in PBS, constituting the *B. velezensis* pellet. Additionally, 15 mL of the original *B. velezensis* culture was preserved for subsequent use.

Then, 1.5 mL each of the *B. velezensis* pellet, ECPs, and PBS (as a control) were deposited in three separate 50 mL centrifuge tubes (Falcon^®^). Measurements of 0.5 µL of C6 AHLs (10 µg/µL) or 0.2 µL of C12 AHLs (10 µg/µL) were added to each Falcon tube. In parallel, 5 µL of C6 AHLs (10 µg/µL) or 2 µL of C6 AHLs (10 µg/µL) was introduced into the preserved 15 mL *B. velezensis* culture. All Falcon tubes were cultured overnight at 26 °C with continuous agitation at 140 rpm. Following the presumed degradation, the resulting cultures were transferred to 1.5 mL tubes and were centrifuged (14,000 rpm, 15 min) to eliminate bacteria. In addition, 10 mL of the presumed degraded *B. velezensis* cultures were employed for pH reconstitution, as described below.

Subsequently, 100 µL of the *B. velezensis* culture, the *B. velezensis* pellet, ECPs, PBS, and AHLs were individually added to separate wells of a 6-well plate containing soft agar (0.4%) with the biomarkers *C. violaceum* CV026 for short-chain AHLs and *C. violaceum* VIR24 for long-chain AHLs. Six-well plates were cultured overnight at 26 °C for 48 h. The entire experiment was conducted in triplicate to ensure the reliability of the results.

### 2.4. AHL Reconstitution via pH Adjustment

The pH reconstitution process was adapted from the methodology outlined by Santos et al. [29] and Singh et al. [30]. The overnight cultures resulting from the interaction between *B. velezensis* D-18 and the respective AHLs were subjected to centrifugation as part of the previously described AHL degradation assay to avoid probiotic bacteria. Aliquots measuring 100 mL of both presumed degraded cultures were utilized in the previously described degradation assay, while the remainder was allocated for the pH reconstitution assay.

Supernatants were adjusted to pH 2 using hydrochloric acid (HCl). Then, 100 µL aliquots of both pH 2 supernatants were carefully added to the wells of the previously prepared 6-well plates containing soft agar (0.4%) with the respective biomarkers, *C. violaceum* CV026 and VIR24. The plates were incubated for 48 h at 26 °C.

### 2.5. PCR Genetic Analysis

Genomic DNA from *B. velezensis* D-18, *B. subtilis* subsp. *subtilis* CECT39, and *B. cereus* CECT148 was extracted and purified using the GeneJET genomic DNA isolation kit (Thermo Scientific, Waltham, MA, USA) to ascertain the presence of lactonase-producing genes within the genome of *B. velezensis* D-18. The experiment utilized specific primers for aiiA (Fw 5′-CGGAATTCATGACAGTAAAGAAGCTTTA-3′; Rv 5′-CGCTCGAGTATATATTCAGGGAACACTT-3′) [31,32] and ytnP (Fw 5′-ATCGGATAATCATCGTAAGC-3′; Rv 5′-ATTGAACTAAGAACAGACCC-3′) [29], which are considered lactonase producer genes. *B. cereus* CECT148 served as the aiiA control, while *B. subtilis* subsp. *subtilis* CECT39 functioned as the ytnP control.

PCR amplification was conducted in a Mastercycler pro S thermal cycler (Eppendorf, Hamburg, Germany) using a 50 μL reaction mixture comprising 5 μL of Taq PCR buffer (10×), 3 μL of MgCl2 (50 mM), 0.2 μL each of 2′-deoxynucleoside 5′-triphosphates (dNTPS) (25 mM), 1 μL of each forward and reverse primer (1:10 dilution), 0.25 μL of DreamTaq DNA polymerase 5 U/μL (Thermo Scientific), and 4 μL of genomic DNA. The PCR conditions included initial denaturation at 95 °C for 1 min followed by 35 cycles of denaturation at 95 °C for 30 s, annealing at 55 °C for 30 s, and extension at 72 °C for 10 s.

Post-PCR cleanup was accomplished using the ExoSAP-IT enzymatic system to eliminate unincorporated primers and dNTPs. Electrophoresis involved the use of diluted 1/10 PCR products, a 2% agarose gel, and GelRed^®^ Nucleic Acid Gel Stain (Biotium, San Francisco, CA, USA) and was conducted under conditions of 80 V for 60 min. The marker employed for reference was the DL2000 Plus DNA Marker.

### 2.6. Vibrio anguillarum 507 Quorum Sensing Signaling Molecules

To elucidate the QS mechanisms of *V. anguillarum* 507, an assay was conducted employing both short-chain and long-chain AHL biomarkers, *C. violaceum* CV026, and VIR24. These biomarkers were separately incorporated into liquid 0.4% LB agar and evenly spread onto Petri dishes. Following solidification, three wells were established in each plate. Subsequently, 10 µL of C12AHL (1 µg/µL), serving as the positive control; 10 µL of PBS, serving as the negative control; 10 µL of an overnight culture of *V. anguillarum* 507 were added to each respective well. The plates were then incubated overnight at 26 °C. The experiment was replicated three times to ensure assay reproducibility.

### 2.7. Bacillus velezensis D-18 Quorum Quenching Effects on Vibrio anguillarum 507

An effective assay was conducted to assess the potential QQ effects of *B. velezensis* D-18 on the marine pathogenic strain *V. anguillarum* 507. Briefly, the probiotic strain, the pathogenic strain, and the long-chain AHL biomarker *C. violaceum* VIR24 were cultured at 26 °C and 140 rpm overnight. Subsequently, 1 mL of *C. violaceum* VIR24 and another mL of *V. anguillarum* were added to 48 mL of 0.4% soft LB agar. This agar was spread onto a Petri dish. Once solidified, 10 µL of the probiotic strain was added to the center of the plate. The plate was then incubated at 26 °C for 24 h. The experiment was performed in triplicate to ensure experimental repeatability.

### 2.8. Inhibition of Biofilm Formation and Growth of Vibrio anguillarum 507 by Bacillus velezensis D-18

After monitoring the growth dynamics of the probiotic bacteria and the pathogen, we assessed the capacity to inhibit biofilm formation and growth through the following procedure. *B. velezensis* was subjected to a 12-h incubation at 37 °C and 140 rpm in 20 mL of BHI supplemented with 1.5% NaCl, yielding a concentration of 10^8^ CFU/mL. Simultaneously, the pathogen *V. anguillarum* was cultivated at 26 °C and 140 rpm for 3 h in 20 mL of BHI supplemented with 1.5% NaCl, resulting in a concentration of 10^7^ CFU/mL. Serial dilutions were conducted to validate these concentrations.

A 1 mL sample was collected from each culture and subjected to centrifugation. The supernatant was removed and then resuspended in 100 µL of sterile PBS. A 12-well plate was employed in the experiment, and an enriched and filtered medium inspired by O’Toole [33] (using BHI supplemented with 1.5% NaCl instead of LB) was prepared to facilitate biofilm formation for both species.

Different well compositions were devised as follows in order to establish the desired concentration of each bacterium for assessing the biofilm formation and growth of both strains. The first comprised 2895 µL of enriched medium, 100 µL of *B. velezensis* (final concentration: 10^8^ CFU/mL), and 5 µL of *V. anguillarum* (final concentration: 10^5^ CFU/mL). The second consisted of 2900 µL of enriched medium and 100 µL of *B. velezensis* (final concentration: 10^8^ CFU/mL). The third included 2995 µL of medium and 5 µL of *V. anguillarum* (final concentration: 10^5^ CFU/mL). The last only included 3000 µL of medium and served as a control. The remaining wells of the 12-well plate were used as controls of the different treatments (*B. velezensis* and *V. anguillarum*, *B. velezensis*, *V. anguillarum*, and control) to confirm biofilm formation using crystal violet (0.1%) after incubation.

The plate was cultured at 26 °C and 100 rpm for 48 h. After this incubation period, the supernatant was removed, and 1 mL from each well was saved for quantification through serial dilutions. Each well underwent three washes with sterile PBS. The well surfaces were scraped, and the material was resuspended in 1 mL of PBS for further serial dilutions to quantify the biofilm amount in UFC/mL.

Serial dilutions of both biofilm formation and culture growth were plated on oxytetracycline (180 μg/mL) plates to quantify the selective growth of *B. velezensis* and on lincomycin (80 μg/mL) plates for *V. anguillarum*. The plates were incubated at 26 °C overnight. This entire experiment was conducted in triplicate to ensure the robustness and reliability of the results.

### 2.9. Statistical Analysis

Statistical analyses were performed using GraphPad Prism software version 8.4.2 for macOS (GraphPad Software, San Diego, CA, USA). The unpaired *t*-test was used to test the differences between the groups. *p* < 0.0001 was defined as statistical significance for all tests that necessitated statistical analyses.

## 3. Results

### 3.1. Quorum Quenching Assay

**Figure 1:**
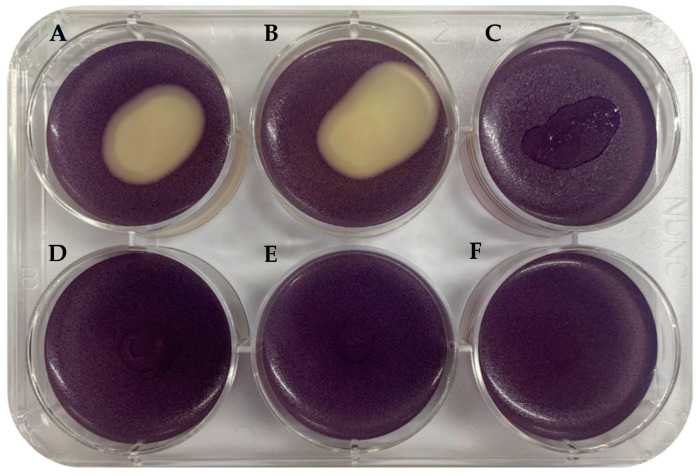
Quorum quenching assay of *Bacillus velezensis* D-18 and its products. *Chromobacterium violaceum* MK produces QS-dependent purple pigment violacein. The lack of production of violacein indicates QQ. (**A**) *B. velezensis* culture. (**B**) *B. velezensis* pellet. (**C**) ECPs. (**D**) Heat-inactivated *B. velezensis*. (**E**) Heat-inactivated ECPs. (**F**) PBS.

The synthesis of violacein by *C. violaceum* MK is a consequence of QS. The QS inhibition by *B. velezensis* (culture and pellet) exhibits an opaque coloration (Figure 1). Importantly, no discernible inhibition was observed in wells containing heat-inactivated *B. velezensis*, PBS, ECPs, heat-inactivated ECPs, and PBS (Figure 1).

### 3.2. AHL Degradation by Bacillus velezensis D-18 and AHL Reconstitution via pH Adjustment

**Figure 2:**
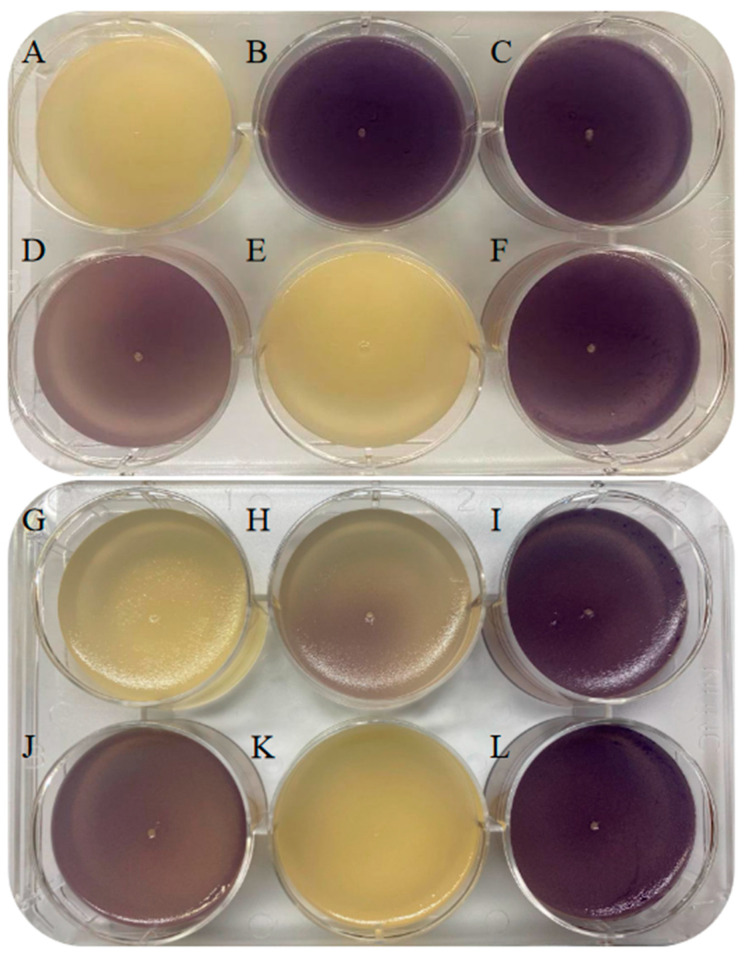
C6 AHL and C12 AHL degradation assay and reconstitution via pH adjustment. C6 AHL degradation assay. (**A**) *B*. *velezensis* culture. (**B**) *B*. *velezensis* pellet. (**C**) ECPs of *B*. *velezensis*. (**D**) Restoration of C6AHL degradation by *B. velezensis* through pH modification. (**E**) PBS. (**F**) C6AHL. C12 AHL degradation assay. (**G**) *B*. *velezensis* culture. (**H**) *B*. *velezensis* pellet. (**I**) ECPs of *B*. *velezensis*. (**J**) Restoration of C12AHL degradation by *B. velezensis* D-18 through pH modification. (**K**) PBS. (**L**) C12AHL.

Following 48 h of growth at 26 °C, the results for the degradation of short-chain AHLs distinctly revealed that wells containing the *B. velezensis* pellet, ECPs, and PBS did not inhibit purple pigment production (Figure 2C,E,F). This implies the absence of inhibitors for short-chain AHL (C6 AHL) components in these conditions. However, the notable inhibition of violacein was observed in the well containing the *B. velezensis* culture (Figure 2A), indicating C6AHL degradation. 

Regarding the degradation of long-chain AHLs, no pigment production was observed using *C. violaceum* VIR24 after 48 h in the *B. velezensis* culture well (Figure 2G). The partial degradation of C12AHL was observed in the well containing the *B. velezensis* pellet (Figure 2H). Nevertheless, violacein production was noted in the well containing ECPs, confirming the absence of degradation (Figure 2I). 

ECPs previously isolated for both AHL degradation assays to test any possible effects on *C. violaceum* CV026 and VIR24 did not interfere in the production of violacein by both biomarkers (Supplementary Figure S2).

AHL reconstitution via the pH technique serves as a valuable tool for identifying degrading enzymes. This method distinguishes between enzymatic degradation by lactonases, which can be reversed under acidic pH conditions, and by acylases which cannot. Next, 10 mL of both short- and long-chain AHLs degraded by the *B. velezensis* culture were presented to the respective biomarkers, *C. violaceum* CV026 and VIR24. After 48 h, violacein production occurred, confirming the successful reconstitution of short- and long-chain AHLs (Figure 2D,J).

### 3.3. Genetic Analysis

**Figure 3:**
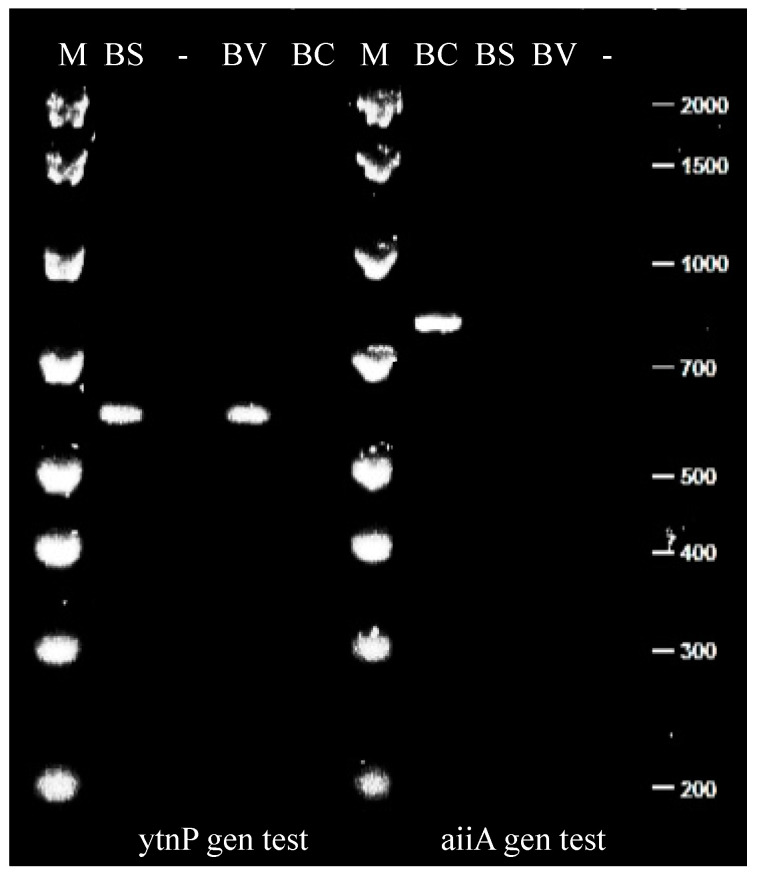
Gene analysis. *B. velezensis* D-18 (BV) DNA was extracted using a commercial kit, and the lactonase genes ytnP (559 bp) and aiiA (756 bp) were tested using PCR. DNA of *B. subtilis* (BS) and *B. cereus* (BC) was used as a control for ytnP and aiiA genes, respectively. (M) DL2000 Marker, (-) Milli-Q water.

Conventional PCR and electrophoresis were conducted to confirm the existence of lactonase genes and compare the in vitro findings. The examination of the results on a 2% agarose gel confirmed the absence of the aiiA gene (756 bp) in the probiotic *B. velezensis* D-18. However, *B. velezensis* D-18 exhibited the presence of the homologous lactonase gene ytnP (559 bp) (Figure 3). The identification of lactonase-producing genes justifies the outcomes observed in the AHL reconstitution via acidic pH.

### 3.4. Vibrio anguillarum 507 Quorum Sensing Signaling Molecules

**Figure 4:**
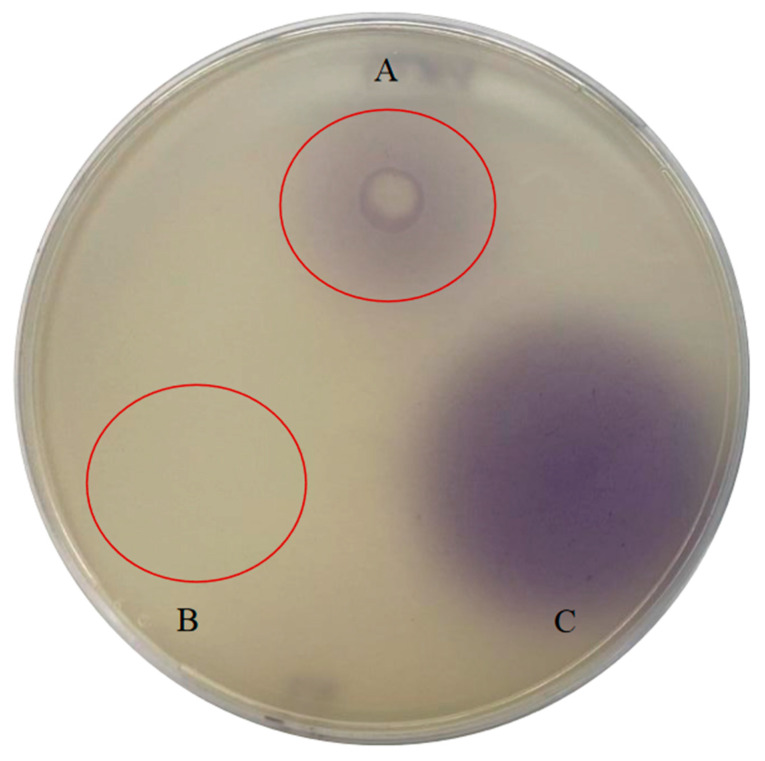
Assay for the demonstration of the presence of long-chain AHLs (QS signaling molecules) via *Vibrio anguillarum* 507. The biomarker *C. violaceum* VIR24 embedded in 0.4% soft LB agar produces violacein pigment upon detecting long-chain AHL molecules. (**A**) *V. anguillarum* 507. (**B**) PBS. (**C**) C12AHL.

In this experimental study, conspicuous evidence of violacein production, indicative of QS signals, was exclusively observed in the 10 µL *V. anguillarum* 507 overnight culture on the *C. violaceum* VIR24 plate (Figure 4A). Conversely, no discernible violacein signals were detected in the plate containing *C. violaceum* CV026 (Supplementary Figure S3). These observations strongly imply the targeted liberation of long-chain AHLs by V. *anguillarum* 507, thereby reaffirming its pivotal involvement in QS within this bacterial strain.

### 3.5. Bacillus velezensis D-18 Quorum Quenching Effects on Vibrio anguillarum 507

**Figure 5:**
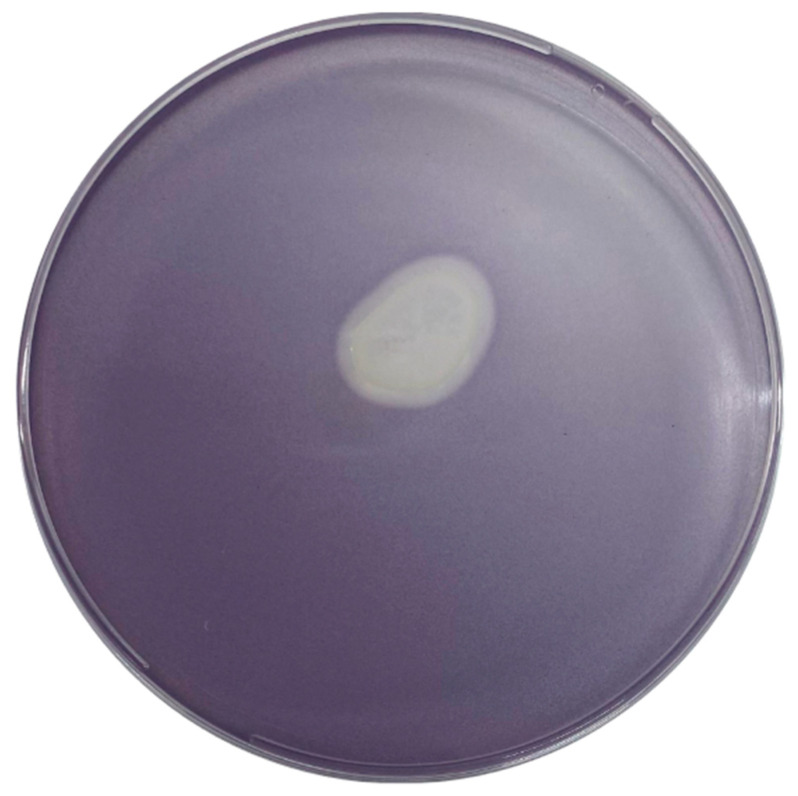
*Bacillus velezensis* D-18 quorum quenching effects on Vibrio anguillarum 507. *B. velezensis* presented to 0.4% LB agar with *V. anguillarum* 507 and *C. violaceum* VIR24. The inhibition halo generated by *B. velezensis* exhibits an opaque coloration, indicative of QQ activity.

Following the incubation of the plate in which *V. anguillarum* was imbibed with the biomarker *C. violaceum* VIR24 with 10 µL of the probiotic added to its surface, an inhibition zone was observed, indicating QQ activity. *C. violaceum* VIR24 did not produce violacein due to the absence of AHL molecules, attributed to the presence of the probiotic (Figure 5).

### 3.6. Inhibition of Biofilm Formation and Growth of Vibrio anguillarum 507 by Bacillus velezensis D-18

**Figure 6:**
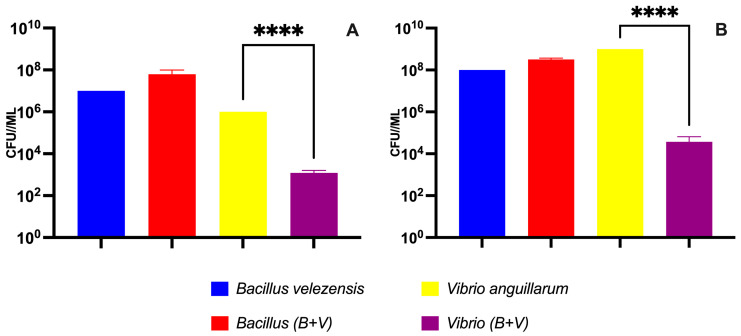
Inhibition of biofilm formation and growth of *Vibrio anguillarum* 507 by *Bacillus velezensis* D-18. (**A**) Biofilm formation following a 48-h co-cultivation of *B. velezensis* (10^8^ CFU/mL) and *V. anguillarum* (10^5^ CFU/mL), denoted as “B + V”. Solitary cultures of *B. velezensis* and *V. anguillarum* were employed as controls. *Bacillus* (B + V) indicates the biofilm formation of *B. velezensis* in the co-culture. *Vibrio* (B + V) indicates the biofilm formation of *V. anguillarum* in the co-culture. (**B**) Bacterial culture after 48 h of co-cultivation of *B. velezensis* (10^8^ CFU/mL) and *V. anguillarum* (10^5^ CFU/mL), denoted as “B + V”. Controls included individual cultures of *B. velezensis* and *V. anguillarum*. *Bacillus* (B + V) indicates the culture growth of *B. velezensis* in the co-culture. *Vibrio* (B + V) indicates the culture growth of *V. anguillarum* in the co-culture. Student’s t-test was used to examine differences in all the parameters tested. Asterisks indicate a significant statistical difference, **** *p* < 0.0001.

Following a 48-h co-culture of probiotic bacteria and the pathogen, the quantification of biofilm formation and culture growth was conducted in terms of CFU/mL. The evaluation of biofilm formation in the co-culture of *B. velezensis* and *V. anguillarum* compared to the control revealed that the presence of the pathogen did not influence *B. velezensis*. The biofilm formation via *B. velezensis* remained comparable to the control, evidencing the probiotic’s robust culture. Conversely, the introduction of the probiotic significantly impacted the biofilm formation of *V. anguillarum* (10^3^ CFU/mL) in contrast to the control, demonstrating the pathogen’s solitary culture (10^6^ CFU/mL) (Figure 6A). The assessment of Bacillus velezensis D-18 and Vibrio anguillarum 507 biofilm formation in control wells stained with 0.1% crystal violet is depicted in Supplementary Figure S4.

The culture growth exhibited analogous results. The co-culture data clearly indicate that the growth of *B. velezensis* was unaffected by the presence of *V. anguillarum*. However, the growth of *V. anguillarum* in the presence of the probiotic was significantly inhibited in comparison to the pathogen control (Figure 6B).

## 4. Discussion

Traditional methods, such as the use of antibiotics, can have detrimental effects on health by fostering the growth of multidrug-resistant bacteria. Therefore, there is an escalating focus on investigating alternative strategies [34]. As described earlier, bacteria employ a signaling process to communicate with each other or with the environment: QS. QS in bacteria is responsible for regulating various processes, including virulence, biofilm formation, sporulation, and the production of secondary metabolites [30,35]. Consequently, QQ, which is the enzymatic disruption of this communication, is emerging as a novel mechanism for bacterial control [19,36].

The application of probiotics with QQ capabilities has recently been on the rise. In this study, we assessed the QQ capacity of the probiotic *B. velezensis* D-18 using a co-cultivation technique with a specific biomarker, *C. violaceum* MK, which produces violacein in response to QS [20]. The disappearance of the purple pigment in *C. violaceum* MK indicated that *B. velezensis* inhibits QS, confirming its QQ ability [30]. Live *B. velezensis* D-18 showed QQ activity, while heat-inactivated *B. velezensis*, ECPs, and heat-inactivated ECPs did not produce an inhibition halo (Figure 2). This suggests that the QQ capacity is associated with live *B. velezensis*, emphasizing the importance of understanding the specific QQ mechanisms involved.

To explore the *B. velezensis* D-18 QQ mechanism, we investigated the degradation of AHLs, the main QS molecules of Gram-negative bacteria, using both short (C6) and long (C12) chains [18]. AHL-producing and AHL-degrading bacteria coexist and employ contrasting strategies to gain a competitive edge over each other [37]. Enzymes catalyzing AHLs can primarily be divided into two groups: (i) those that lead to the degradation of the homoserine lactone ring, known as lactonases, and (ii) those that cause cleavage in the bond between the acyl chain and the homoserine lactone, known as acylases [19]. In the case of acylases, enzymatic degradation is conditioned by the length of the carbon rings, making it highly specific. However, lactonases interact directly with AHLs [19,38]. The *Bacillus* genus is known to degrade AHLs due to the presence of genes that produce degrading enzymes [19,38]. Therefore, the *B. velezensis* culture, the *B. velezensis* pellet, and ECPs were utilized to assess the degradation of long- (C12) and short-chain (C6) AHLs using *C. violaceum* VIR24 and CV026 as biomarkers, respectively [39]. The study revealed that the *B. velezensis* D-18 culture was responsible for the degradation of both forms of AHLs. The *B. velezensis* D-18 pellet exhibited partial degradation of C12AHL. However, it was unable to degrade C6AHL. ECPs showed no capability to degrade any AHL molecules, which contrasts with previous research highlighting the extracellular QQ potential of *Bacillus* spp. mediated by ECPs [29]. Nevertheless, other studies on probiotic candidates have demonstrated intracellular QQ activity [21]. In this study, the majority or sole degradation occurred with the *B. velezensis* culture. There is a strong indication of the presence of an inducible lactonase producer gene in *B. velezensis* D-18 and its subsequent release in the presence of AHL molecules during the biological development of the bacteria. This hypothesis is supported by the well-established communication among bacteria through the emission and uptake of autoinducers (AIs). As previously described, Gram-positive bacteria QS is mediated by autoinducer peptides (AIPs), which are typically released extracellularly and are, therefore, present in the surrounding medium. Gram-positive bacteria can detect and respond to AIPs to regulate their metabolic activities [15]. This feedback loop involving AIPs may enhance QS activity, leading to gene regulation that potentially triggers higher QQ activity, resulting in the detection of AHL molecules and the increase in degradation by *B. velezensis* D-18.

Consequently, the degradation of both long- and short-chain AHLs strongly suggested the presence of lactonase activity. To confirm this, these degraded AHLs were subjected to pH reduction and then exposed to the respective biomarkers. This resulted in the production of violacein, indicating the reconstitution of AHLs. Therefore, the enzyme was confirmed to be a lactonase. Researchers have argued that acylase enzymes have more advantages in practical applications since the AHLs degraded by lactonase could be reconstituted by lowering the pH [17,40]. However, lactonases have a wide range of effects on long- and short-chain AHLs, unlike acylases, which are generally most effective against AHLs with side chains longer than 10 carbon atoms [16,40].

We verified the presence of the lactonase-producing gene in *Bacillus velezensis* D-18 despite confirming that the enzymatic reaction is performed by a lactonase. Several authors support that the aiiA lactonase producer gene is inherent to numerous *Bacillus* spp. [19,25,32]. Recently, researchers have demonstrated the presence of homologous lactonase-producing genes in several *Bacillus* spp., such as the ytnP gene [41]. In this study, PCR was conducted using primers designed and used by previous researchers [29,31,32] to determine the AHL-producing genes of the probiotic. The PCR results confirmed the absence of the aiiA gene in *B. velezensis* D-18, which aligns with findings in other *Bacillus* spp. [29,32]. However, PCR results confirmed the presence of the homologous lactonase gene ytnP (584 bp) in *B. velezensis* D-18, suggesting an alternative mechanism for QQ activity in accordance with previous studies that identified homologous lactonase-producing genes in *Bacillus* species [25,29,32].

As previously mentioned, pathogenic bacteria form biofilms to adapt to environmental conditions and evade antibacterial agents. This bacterial protection mechanism has led to a decrease in the effectiveness of antibiotic treatments [42]. In the field of aquaculture, biofilms serve as significant reservoirs for pathogenic microorganisms. In particular, the presence of *Vibrio* spp. in aquaculture systems is the cause of numerous economic losses, making its control essential [36,43]. Therefore, the reduction and control of *Vibrio* biofilms contribute to an improvement in animal welfare. Several researchers have offered differing perspectives on the implication of QS in biofilm formation [12,18,42]. QS is one of the mechanisms responsible for many biofilm stages such as bacterial adhesion, biofilm production, and bacterial dispersion [44]. The presence of AIs is also crucial for biofilm formation. In Gram-negative bacteria, AHLs play a crucial role in biofilm development and dispersion [45]. Throughout this study, we have verified that *Vibrio anguillarum* 507 releases long-chain AHLs as QS signal molecules. Numerous investigations have previously delineated the regulatory mechanisms of AHLs in Vibrio spp. and their roles in biofilm formation [12,46,47]. Drawing upon the documented QQ effects of *Bacillus* spp. on *Vibrio* biofilms [48,49], we explored the potential QQ impacts of *B. velezensis* D-18 on *V. anguillarum* 507.

This study unveiled that *B. velezensis* D-18 exerts QQ effects on *V. anguillarum* by degrading AHLs. This was evidenced by the absence of violacein production when the long-chain AHL biomarker *C. violaceum* VIR24 was present. In subsequent assays, the formation of *B. velezensis* D-18 biofilms remained unaffected by the presence of the pathogen *V. anguillarum*. Conversely, the presence of *B. velezensis* significantly impacted the biofilm formation of *V. anguillarum*, suggesting its potential as a biofilm control agent. These findings were mirrored in culture growth, with *B. velezensis* thriving in the co-culture, while the growth of *V. anguillarum* was notably reduced. The evident inhibition of QS in *V. anguillarum* 507, characterized by a lack of observable growth in the medium without a decrease in colonies, underscores the impactful role of *B. velezensis* QQ. The production of lactonase by the probiotic serves as a pivotal factor, actively degrading the signaling molecules, AHLs, crucial for the communication network of the pathogen. This disruption deprives *V. anguillarum* of the vital communication needed to initiate gene expression related to virulence factors, biofilm formation, and growth [19]. Our findings shed light on the multifaceted QQ mechanisms employed by *B. velezensis* D-18. The presence of ytnP further contributes to our understanding of the diverse QQ strategies employed by *Bacillus* species. These results reinforce the position of *Bacillus* spp. as promising candidates for preventing *Vibrio* diseases in aquaculture due to their comprehensive QQ capabilities [24,50].

## 5. Conclusions

In conclusion, *Bacillus velezensis* D-18 presents a promising avenue for the prevention of *Vibrio anguillarum* 507 diseases in aquaculture due to its quorum quenching capacity as an enzymatic disruptor of AHLs. The ability to disrupt bacterial communication and control biofilm formation positions *B. velezensis* D-18 as a potential eco-friendly alternative to conventional antibiotics in managing bacterial diseases in aquaculture.

## Data Availability

Data are contained within the article (and Supplementary Materials).

## Supplementary Materials

The following supporting information can be downloaded at: https://www.mdpi.com/article/10.3390/microorganisms12050890/s1, Figure S1: Any interference by *Bacillus velezensis* D-18 Extracellular Products (ECPs) on violacein production by *C. violaceum* CV026. (A). ECPs. (B). Heat-inactivated ECPs. (C). C6AHL (1 ug/uL), Figure S2: Any interference by *Bacillus velezensis* D-18 Extracellular Products (ECPs) on violacein production by *C. violaceum* VIR24. (A). ECPs. (B). Heat-inactivated ECPs. (C). C6AHL (1 ug/uL), Figure S3: Assay for the demonstration of the presence of QS signaling molecules by *Vibrio anguillarum* 507. The biomarker *C. violaceum* CV026, embedded in 0.4% soft LB agar, produces violacein pigment upon detecting short-chains AHL molecules. (A) *Vibrio anguillarum* 507. (B) PBS. (C) C6AHL (1 ug/uL), Figure S4: *Bacillus velezensis* D-18 and *Vibrio anguillarum* 507 biofilms stained with 0.1% Crystal Violet (CV).

## References

[1] Arechavala-Lopez P., Cabrera-Álvarez M.J., Maia C.M., Saraiva J.L. (2022). Environmental Enrichment in Fish Aquaculture: A Review of Fundamental and Practical Aspects. Rev. Aquac..

[2] Yada T., Tort L. (2016). Stress and Disease Resistance: Immune System and Immunoendocrine Interactions. Fish Physiol..

[3] Gao X.Y., Liu Y., Miao L.L., Li E.W., Hou T.T., Liu Z.P. (2017). Mechanism of Anti-*Vibrio* Activity of Marine Probiotic Strain *Bacillus pumilus* H2, and Characterization of the Active Substance. AMB Express.

[4] Frans I., Michiels C.W., Bossier P., Willems K.A., Lievens B., Rediers H. (2011). *Vibrio anguillarum* as a Fish Pathogen: Virulence Factors, Diagnosis and Prevention. J. Fish Dis..

[5] Arunkumar M., LewisOscar F., Thajuddin N., Pugazhendhi A., Nithya C. (2020). In Vitro and In Vivo Biofilm Forming *Vibrio* spp: A Significant Threat in Aquaculture. Process Biochem..

[6] Flemming H.C., Wingender J., Szewzyk U., Steinberg P., Rice S.A., Kjelleberg S. (2016). Biofilms: An Emergent Form of Bacterial Life. Nat. Rev. Microbiol..

[7] Brackman G., Coenye T. (2015). Quorum Sensing Inhibitors as Anti-Biofilm Agents. Curr. Pharm. Des..

[8] Wang Y., Bian Z., Wang Y. (2022). Biofilm Formation and Inhibition Mediated by Bacterial Quorum Sensing. Appl. Microbiol. Biotechnol..

[9] Nikaido H. (2009). Multidrug Resistance in Bacteria. Annu. Rev. Biochem..

[10] Yi L., Dong X., Grenier D., Wang K., Wang Y. (2021). Research Progress of Bacterial Quorum Sensing Receptors: Classification, Structure, Function and Characteristics. Sci. Total Environ..

[11] Zhong S., He S. (2021). Quorum Sensing Inhibition or Quenching in Acinetobacter Baumannii: The Novel Therapeutic Strategies for New Drug Development. Front. Microbiol..

[12] García-Aljaro C., Melado-Rovira S., Milton D.L., Blanch A.R. (2012). Quorum-Sensing Regulates Biofilm Formation in *Vibrio scophthalmi*. BMC Microbiol..

[13] Pena R.T., Blasco L., Ambroa A., González-Pedrajo B., Fernández-García L., López M., Bleriot I., Bou G., García-Contreras R., Wood T.K. (2019). Relationship between Quorum Sensing and Secretion Systems. Front. Microbiol..

[14] Dhiman S.S. (2020). Introduction to Quorum Sensing. ACS Symp. Ser..

[15] Wu S., Liu J., Liu C., Yang A., Qiao J. (2020). Quorum Sensing for Population-Level Control of Bacteria and Potential Therapeutic Applications. Cell. Mol. Life Sci..

[16] Sikdar R., Elias M. (2020). Quorum Quenching Enzymes and Their Effects on Virulence, Biofilm, and Microbiomes: A Review of Recent Advances. Expert Rev. Anti Infect. Ther..

[17] Shen Y., Cui F., Wang D., Li T., Li J. (2021). Quorum Quenching Enzyme (Pf-1240) Capable to Degrade Ahls as a Candidate for Inhibiting Quorum Sensing in Food Spoilage Bacterium *Hafnia alvei*. Foods.

[18] Paluch E., Rewak-Soroczyńska J., Jędrusik I., Mazurkiewicz E., Jermakow K. (2020). Prevention of Biofilm Formation by Quorum Quenching. Appl. Microbiol. Biotechnol..

[19] Chen F., Gao Y., Chen X., Yu Z., Li X. (2013). Quorum Quenching Enzymes and Their Application in Degrading Signal Molecules to Block Quorum Sensing-Dependent Infection. Int. J. Mol. Sci..

[20] Singh A.A., Singh A.K., Nerurkar A. (2021). Disrupting the Quorum Sensing Mediated Virulence in Soft Rot Causing *Pectobacterium carotovorum* by Marine Sponge Associated *Bacillus* sp. OA10. World J. Microbiol. Biotechnol..

[21] Rehman Z.U., Leiknes T.O. (2018). Quorum-Quenching Bacteria Isolated from Red Sea Sediments Reduce Biofilm Formation by *Pseudomonas aeruginosa*. Front. Microbiol..

[22] Hill C., Guarner F., Reid G., Gibson G.R., Merenstein D.J., Pot B., Morelli L., Canani R.B., Flint H.J., Salminen S. (2014). The International Scientific Association for Probiotics and Prebiotics Consensus Statement on the Scope and Appropriate Use of the Term Probiotic. Nat. Rev. Gastroenterol. Hepatol..

[23] Monzón-Atienza L., Bravo J., Serradell A., Montero D., Gómez-Mercader A., Acosta F. (2023). Current Status of Probiotics in European Sea Bass Aquaculture as One Important Mediterranean and Atlantic Commercial Species: A Review. Animals.

[24] Kuebutornye F.K., Abarike E.D., Lu Y., Hlordzi V., Essien Sakyi M., Afriyie G., Wang Z., Li Y., Xia C.X. (2020). Mechanisms and the Role of Probiotic *Bacillus* in Mitigating Fish Pathogens in Aquaculture. Fish Physiol. Biochem..

[25] Noor A.O., Almasri D.M., Basyony A.F., Albohy A., Almutairi L.S., Alhammadi S.S., Alkhamisi M.A., Alsharif S.A., Elfaky M.A. (2022). Biodiversity of N-Acyl Homoserine Lactonase (AiiA) Gene from *Bacillus subtilis*. Microb. Pathog..

[26] Rosier A., Beauregard P.B., Bais H.P. (2021). Quorum Quenching Activity of the PGPR Bacillus Subtilis UD1022 Alters Nodulation Efficiency of Sinorhizobium Meliloti on *Medicago truncatula*. Front. Microbiol..

[27] Monzón-Atienza L., Bravo J., Torrecillas S., Montero D., de Canales A.F.G., de la Banda I.G., Galindo-Villegas J., Ramos-Vivas J., Acosta F. (2021). Isolation and Characterization of a *Bacillus* Velezensis D-18 Strain, as a Potential Probiotic in European Seabass Aquaculture. Probiotics Antimicrob. Proteins.

[28] Monzón-Atienza L., Bravo J., Fernández-Montero Á., Charlie-Silva I., Montero D., Ramos-Vivas J., Galindo-Villegas J., Acosta F. (2022). Dietary Supplementation of *Bacillus velezensis* Improves *Vibrio anguillarum* Clearance in European Sea Bass by Activating Essential Innate Immune Mechanisms. Fish Shellfish Immunol..

[29] Santos R.A., Monteiro M., Rangel F., Jerusik R., Saavedra M.J., Carvalho A.P., Oliva-Teles A., Serra C.R. (2021). *Bacillus* spp. Inhibit Edwardsiella Tarda Quorum-Sensing and Fish Infection. Mar. Drugs.

[30] Singh A.A., Singh A.K., Nerurkar A. (2020). Bacteria Associated with Marine Macroorganisms as Potential Source of Quorum-Sensing Antagonists. J. Basic Microbiol..

[31] Nusrat H., Shankar P., Kushwah J., Bhushan A., Joshi J., Mukherjee T., Raju S.C., Purohit H.J., Kalia V.C. (2011). Diversity and Polymorphism in AHL-Lactonase Gene (AiiA) of *Bacillus*. J. Microbiol. Biotechnol..

[32] El Aichar F., Muras A., Parga A., Otero A., Nateche F. (2022). Quorum Quenching and Anti-biofilm Activities of Halotolerant *Bacillus* Strains Isolated in Different Environments in Algeria. J. Appl. Microbiol..

[33] O’Toole G.A. (2011). Microtiter Dish Biofilm Formation Assay. J. Vis. Exp..

[34] Arsene M.M.J., Jorelle A.B.J., Sarra S., Viktorovna P.I., Davares A.K.L., Ingrid N.K.C., Steve A.A.F., Andreevna S.L., Vyacheslavovna Y.N., Carime B.Z. (2021). Short Review on the Potential Alternatives to Antibiotics in the Era of Antibiotic Resistance. J. Appl. Pharm. Sci..

[35] Rutherford S.T., Bassler B.L. (2012). Bacterial Quorum Sensing: Its Role in Virulence and Possibilities for Its Control. Cold Spring Harb. Perspect. Med..

[36] Sun X., Liu J., Deng S., Li R., Lv W., Zhou S., Tang X., Sun Y.Z., Ke M., Wang K. (2022). Quorum Quenching Bacteria *Bacillus* velezensis DH82 on Biological Control of *Vibrio parahaemolyticus* for Sustainable Aquaculture of *Litopenaeus vannamei*. Front. Mar. Sci..

[37] Chen B., Peng M., Tong W., Zhang Q., Song Z. (2020). The Quorum Quenching Bacterium *Bacillus licheniformis* T-1 Protects Zebrafish against *Aeromonas hydrophila* Infection. Probiotics Antimicrob. Proteins.

[38] Fetzner S. (2015). Quorum Quenching Enzymes. J. Biotechnol..

[39] Remuzgo-Martínez S., Lázaro-Díez M., Mayer C., Aranzamendi-Zaldumbide M., Padilla D., Calvo J., Marco F., Martínez-Martínez L., Icardo J.M., Otero A. (2015). Biofilm Formation and Quorum-Sensing-Molecule Production by Clinical Isolates of *Serratia liquefaciens*. Appl. Environ. Microbiol..

[40] Murugayah S.A., Gerth M.L. (2019). Engineering Quorum Quenching Enzymes: Progress and Perspectives. Biochem. Soc. Trans..

[41] Djokic L., Stankovic N., Galic I., Moric I., Radakovic N., Šegan S., Pavic A., Senerovic L. (2022). Novel Quorum Quenching YtnP Lactonase from *Bacillus paralicheniformis* Reduces Pseudomonas Aeruginosa Virulence and Increases Antibiotic Efficacy in Vivo. Front. Microbiol..

[42] Kalia V.C., Patel S.K.S., Lee J.K. (2023). Bacterial Biofilm Inhibitors: An Overview. Ecotoxicol. Environ. Saf..

[43] Sanches-Fernandes G.M.M., Sá-Correia I., Costa R. (2022). Vibriosis Outbreaks in Aquaculture: Addressing Environmental and Public Health Concerns and Preventive Therapies Using Gilthead Seabream Farming as a Model System. Front. Microbiol..

[44] Zhou L., Zhang Y., Ge Y., Zhu X., Pan J. (2020). Regulatory Mechanisms and Promising Applications of Quorum Sensing-Inhibiting Agents in Control of Bacterial Biofilm Formation. Front. Microbiol..

[45] Hu H., He J., Liu J., Yu H., Tang J., Zhang J. (2016). Role of N-Acyl-Homoserine Lactone (AHL) Based Quorum Sensing on Biofilm Formation on Packing Media in Wastewater Treatment Process. RSC Adv..

[46] Liu J., Fu K., Wang Y., Wu C., Li F., Shi L., Ge Y., Zhou L. (2017). Detection of Diverse N-Acyl-Homoserine Lactones in Vibrio Alginolyticus and Regulation of Biofilm Formation by N-(3-Oxodecanoyl) Homoserine Lactone in Vitro. Front. Microbiol..

[47] Liu J., Fu K., Wu C., Qin K., Li F., Zhou L. (2018). “In-Group” Communication in Marine *Vibrio*: A Review of N-Acyl Homoserine Lactones-Driven Quorum Sensing. Front. Cell Infect. Microbiol..

[48] Vinoj G., Vaseeharan B., Thomas S., Spiers A.J., Shanthi S. (2014). Quorum-Quenching Activity of the AHL-Lactonase from *Bacillus licheniformis* DAHB1 Inhibits Vibrio Biofilm Formation In Vitro and Reduces Shrimp Intestinal Colonisation and Mortality. Mar. Biotechnol..

[49] Augustine N., Kumar P., Thomas S. (2010). Inhibition of *Vibrio cholerae* biofilm by AiiA Enzyme Produced from *Bacillus* spp.. Arch. Microbiol..

[50] Shaheer P., Sreejith V.N., Joseph T.C., Murugadas V., Lalitha K.V. (2021). Quorum Quenching *Bacillus* spp.: An Alternative Biocontrol Agent for *Vibrio harveyi* Infection in Aquaculture. Dis. Aquat. Organ..

