# Supplementary material for: An In-Depth Study on the Inhibition of Quorum Sensing by Bacillus velezensis D-18: Its Significant Impact on Vibrio Biofilm Formation in Aquaculture
Source: Microorganisms. 2024 Apr 29;12(5):890. doi: 10.3390/microorganisms12050890 (PMC11123725; doi:10.3390/microorganisms12050890)
Supplement: Supplementary file 1 [file microorganisms-12-00890-s001.zip › microorganisms-2972308-supplementary.pdf]

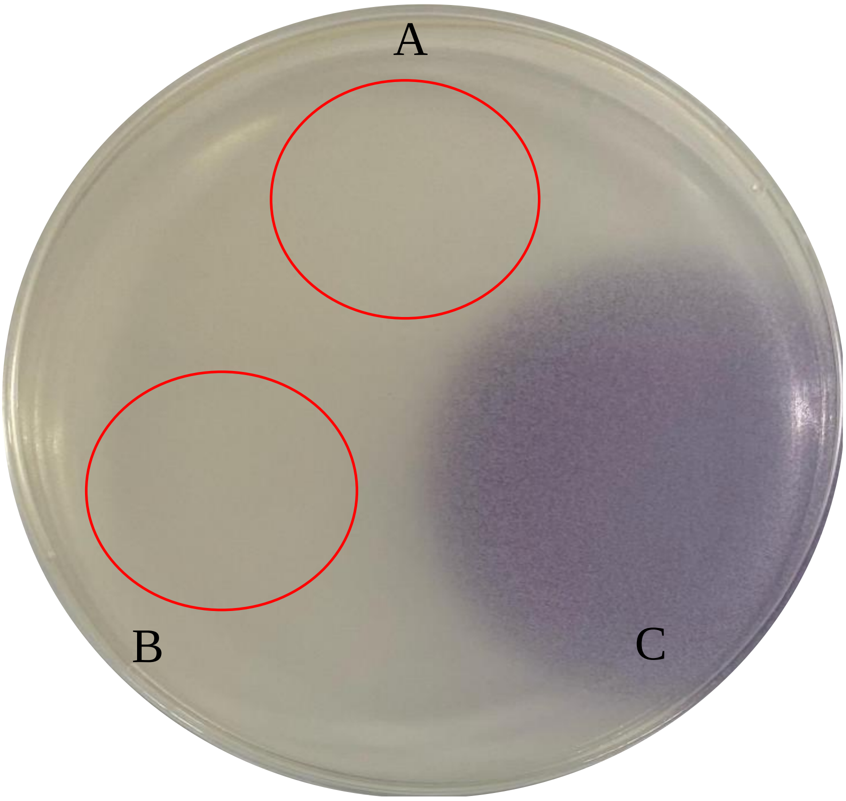

Supplementary Figure S1. Any interference by *Bacillus velezensis* D-18 Extracellular Products (ECPs) on violacein production by *C. violaceum* CV026. A. ECPs. **B**. Heat-inactivated ECPs. **C**. C6AHL (1ug/uL)

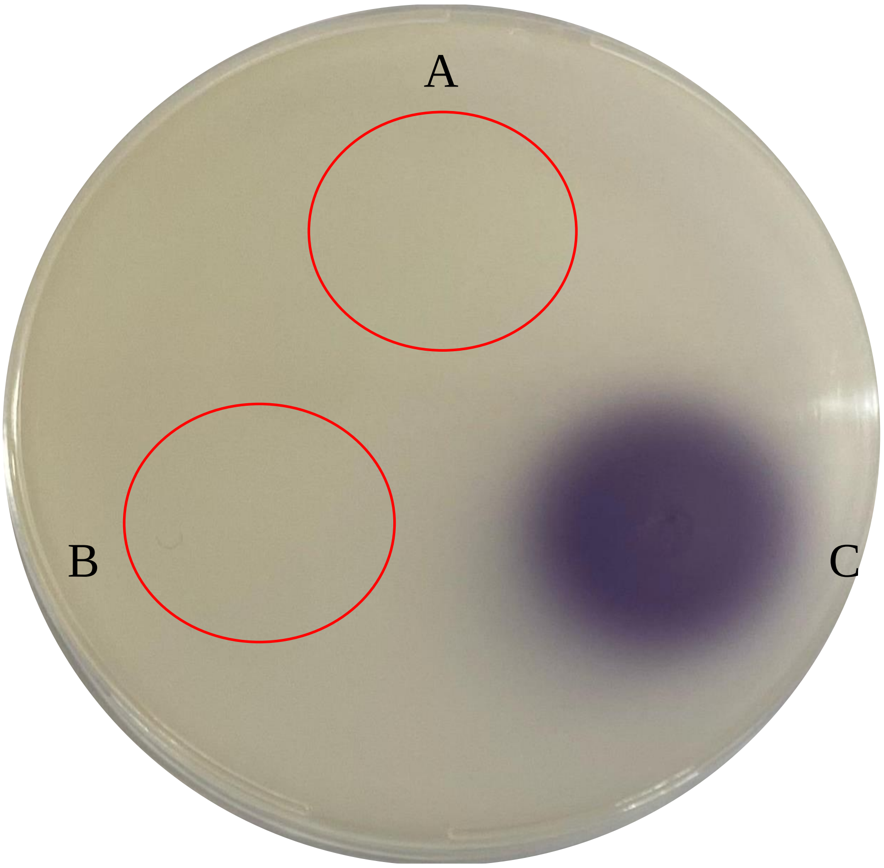

Supplementary Figure S2. Any interference by *Bacillus velezensis* D-18 Extracellular Products (ECPs) on violacein production by *C. violaceum* VIR24. A. ECPs. **B**. Heat-inactivated ECPs. **C**. C6AHL (1ug/uL)

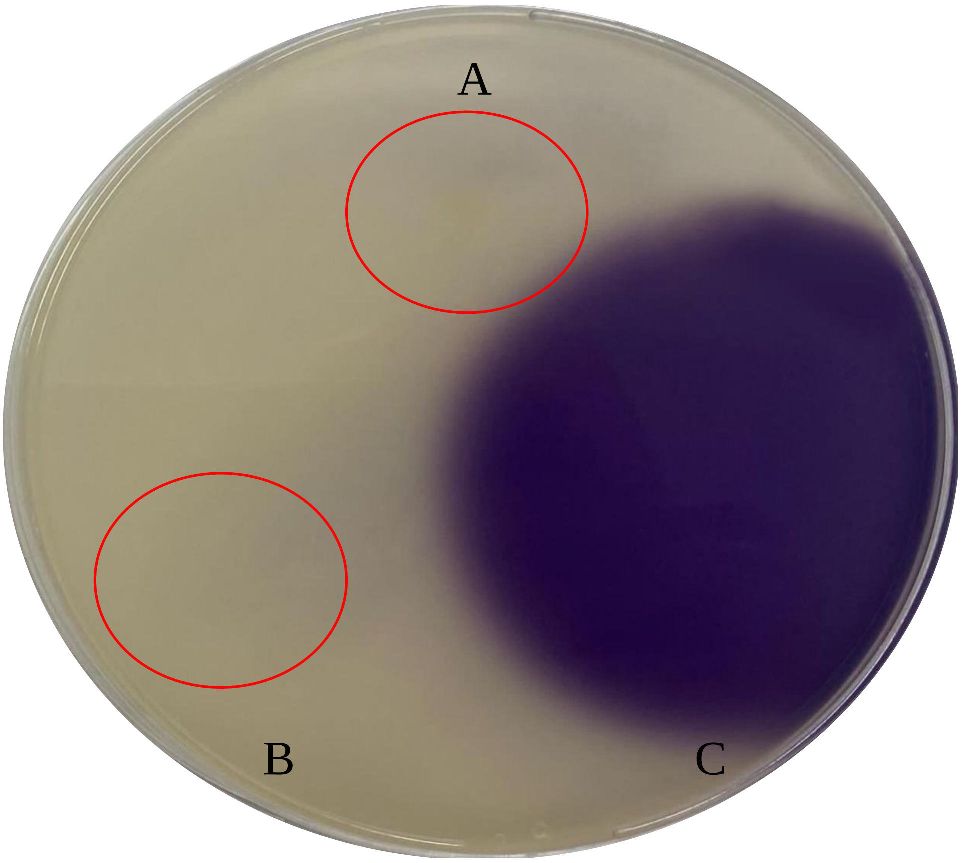

Supplementary Figure S3. Assay for the demonstration of the presence of QS signaling molecules by *Vibrio anguillarum* 507. The biomarker *C. violaceum* CV026, embedded in 0.4% soft LB agar, produces violacein pigment upon detecting short-chains AHL molecules. A) *Vibrio anguillarum* 507. B) PBS. C) C6AHL (1ug/uL).

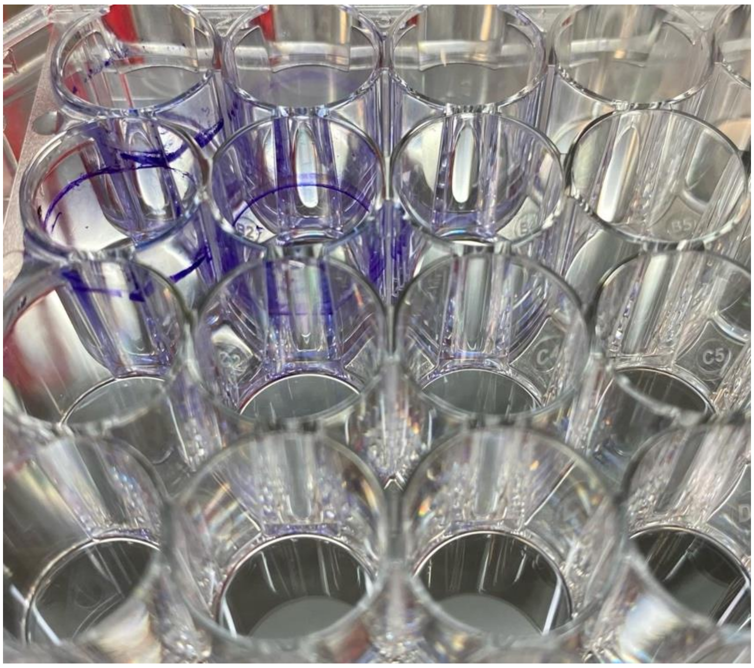

Supplementary Figure S4. *Bacillus velezensis* D-18 and *Vibrio anguillarum* 507 biofilms stained with 0.1% Crystal Violet (CV).
